# Bedside breath tests in children with abdominal pain: a prospective pilot feasibility study

**DOI:** 10.1186/s40814-019-0502-x

**Published:** 2019-11-05

**Authors:** David C. Wong, Samuel D. Relton, Victoria Lane, Mohamed Ismail, Victoria Goss, Jane Bytheway, Robert M. West, Jim Deuchars, Jonathan Sutcliffe

**Affiliations:** 1 Centre for Health Informatics, University of Manchester, Manchester, UK; 2 Leeds Institute of Health Sciences, University of Leeds, Leeds, UK; 3 Leeds Teaching Hospitals Trust, Leeds, UK; 4 Leeds Institute for Clinical Trials Research, University of Leeds, Leeds, UK; 5 Public Contributor, ., USA; 6 School of Biomedical Sciences, University of Leeds, Leeds, UK

**Keywords:** Appendicitis, Child, Exhalation, Volatile organic compounds, Breathomics, Biomarkers

## Abstract

**Background:**

There is no definitive method of accurately diagnosing appendicitis before surgery. We evaluated the feasibility of collecting breath samples in children with abdominal pain and gathered preliminary data on the accuracy of breath tests.

**Methods:**

We conducted a prospective pilot study at a large tertiary referral paediatric hospital in the UK. We recruited 50 participants with suspected appendicitis, aged between 5 and 15 years. Five had primary diagnosis of appendicitis. The primary outcome was the number of breath samples collected. We also measured the number of samples processed within 2 h and had CO_2_ ≥ 3.5%. Usability was assessed by patient-reported pain pre- and post-sampling and user-reported sampling difficulty. Logistic regression analysis was used to predict appendicitis and evaluated using the area under the receiver operator characteristic curve (AUROC).

**Results:**

Samples were collected from all participants. Of the 45 samples, 36 were processed within 2 h. Of the 49 samples, 19 had %CO_2_ ≥ 3.5%. No difference in patient-reported pain was observed (*p* = 0.24). Sampling difficulty was associated with patient age (*p* = 0.004). The logistic regression model had AUROC = 0.86.

**Conclusions:**

Breath tests are feasible and acceptable to patients presenting with abdominal pain in clinical settings. We demonstrated adequate data collection with no evidence of harm to patients. The AUROC was better than a random classifier; more specific sensors are likely to improve diagnostic performance.

**Trial registration:**

ClinicalTrials.gov, NCT03248102. Registered 14 Aug 2017.

## Introduction

Exhaled breath tests from patients have previously been tested as a method to predict respiratory, liver and infectious diseases [1–6]. These tests detect the presence of key volatile organic compounds (VOCs) that provide a unique biomarker for the disease.

Exhaled breath tests may be useful in the diagnosis of common abdominal conditions. For some gastrointestinal conditions including acute appendicitis, halitosis, or fetor, is a commonly reported symptom [7, 8]. Halitosis is thought to be due to the creation of organic compounds that are a byproduct of bacterial infection. Primary diagnosis halitosis has previously been identified using VOC analysis [9].

Acute appendicitis is a common condition in children [10, 11]; however, timely and accurate diagnosis remains challenging despite access to multiple diagnostic modalities. Delayed diagnosis is frequent (reported as being up to 60%) and associated with increases in appendix perforation rate from 21 to 71% [12]. Perforation is associated with significant increases in morbidity, length of stay and cost [13, 14]. False-positive diagnosis leading to unnecessary surgery has been estimated at 10–12% [15, 16]. Delayed diagnosis is thought to be due to variable, non-specific presentation [17]. In children, diagnosis is further complicated by the inability to articulate symptoms.

The possibility of improving the accuracy of appendicitis diagnosis in the paediatric patient population is highly appealing. An exhaled breath test has the potential to be less expensive and invasive than current blood test or imaging diagnostic techniques, especially if it can provide equivalent effectiveness.

The feasibility of exhaled breath tests requires the test to be tolerated by those with suspected appendicitis. The vast majority of these patients present with abdominal pain, and it is currently unknown whether breath tests would exacerbate the pain. In addition, any collected breath data must be of sufficient quality for analysis and must be processed in a timely manner for clinical decision making. In this study, we investigate these feasibility issues. A secondary objective was to obtain preliminary information on the composition of VOCs in children, with and without appendicitis. These data will inform future studies.

## Methods

### Study design

This was a single-centre prospective pilot study conducted in a large tertiary referral paediatric hospital. Approval for this study was obtained from the NHS Research Ethics Committee (REC No. 17/WM/0151) and registered with ClinicalTrials.gov (ID: NCT03248102). Approval for use of all equipment was obtained from the hospital trust’s Medical Physics and Infection Control units.

### Participants

Children aged between 5 and 15 inclusive, presenting with suspected appendicitis, were recruited. These were typically patients who had been referred to the paediatric surgical team after presentation via the Emergency Department or the Children’s Assessment Unit or through direct referral from another team or hospital. Participants came from a non-consecutive convenience sample, based on the availability of the research assistant.

The research assistant (MI) was a medical student who was supervised by a consultant paediatric surgeon (JS). In addition to 24-h access to the consultant, the research assistant had additional support available from two other senior members of the consultant staff and a paediatric surgical registrar (VL). The RA was available during ‘office hours’ Monday to Friday subject to his course commitments. In addition, when available, he collected samples in the evenings and weekends.

Participants were excluded if they had a known alternative cause of abdominal pain (e.g. Crohn’s disease) or if they had been admitted and discharged before a researcher was able to obtain consent.

### Data collection

#### Participant characteristics and clinical data

Baseline characteristics were collected for each participant. These were age in months, sex and admission date and time. The following clinical data were also collected: operation date and time, current medication, current use of antibiotics and histopathological diagnosis.

#### Breath sample data

The research assistant was alerted to the presence of a patient with suspected appendicitis by the clinical team. Initially, they met the patient and their family to discuss the study and provided an age-specific patient and parent/guardian information sheet. Informed written consent was sought from the parent/guardian of the potential participant, and patients were excluded if consent was not provided. A patient or their parent/guardian was able to verbally withdraw from the study during their in-patient stay and via written request up to the point of completion of data analysis.

After consent, a single breath sample was collected from recruited participants via a custom-made mouthpiece attached to a Tedlar® bag primed with 200 μl of distilled water. The process of collecting a breath sample was as follows. The participant was seated and asked to rest for 5 min prior to sampling. The participant was then instructed to take a large breath in and exhale via the mouthpiece. After 4 s, the researcher capped the end of the mouthpiece so that the Tedlar® bag was filled with the end-tidal fraction of breath. The end-tidal, or alveolar, fraction was required to ensure reliable breath composition [18]. Breath samples can be classified as alveolar if the %CO_2_ ≥ 3.5% [19].

Further exhaled breaths were collected in the same bag in the event of insufficient breath volume (as determined by visual inspection). Alongside the breath sample itself, the date and time of the sample, participant pain (scored 0–10) before and after breath collection and difficulty of breath collection (scored 0–10) were also collected. Pain assessment scores between 0 and 10 are common in clinical care [20] and have been validated in paediatric cohorts [21].

#### VOC data

**Fig. 1:**
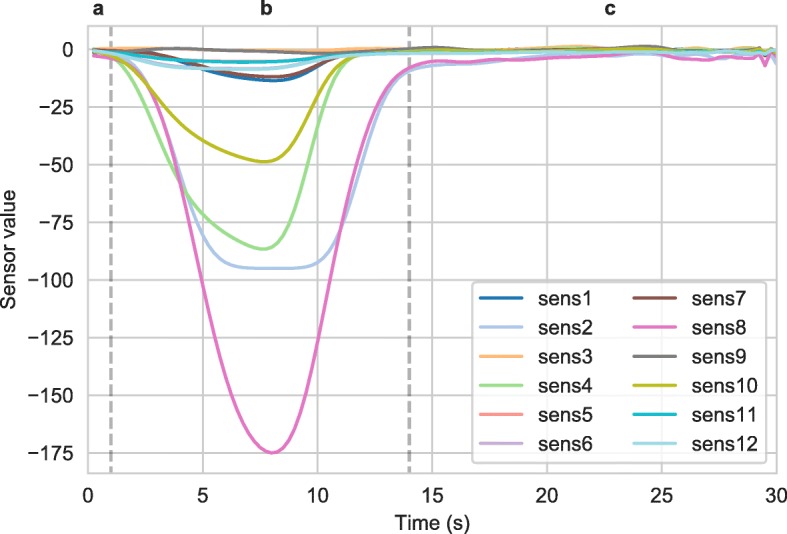
Example of a single VOC signature with 12 sensors. The time captured includes three phases: **a** a small warm-up phase (0–2 s), **b** the sensor readings as the sample is passed through the device (2–13 s) and **c** a post-sample phase consisting of noise in the sensors whilst the device is reset (17–30 s)

Breath samples were transported at room temperature for analysis using a Bloodhound® electronic nose (e-nose) attached to a laptop PC, which contained 12 non-specific sensors. The e-nose equipment was kept in a room adjacent to the paediatric surgical ward, in which room temperature and humidity were monitored. The output of the e-nose is a VOC signature. Each VOC signature is a 30-s 12-channel time series (Fig. 1). The e-nose recording frequency is 4 Hz, leading to 1440 data points per VOC signature. Each sample of breath was repeatedly processed until a consistent VOC signature was obtained, as there is an initial ‘warm-up’ period during which the readings can vary significantly. To reduce the level of noise in the final time series, all analyses were undertaken on the average of the last three signatures for each patient.

### Data storage and cleaning

VOC data were stored electronically and assigned a filename containing the study ID. All other data were collected on paper case report forms and transcribed into an electronic database by SR and DW. In addition, the start and end time of analysis and the %CO_2_ contained in the breath sample were recorded. A total of 5 forms (10%) were randomly selected using MATLAB’s *randperm* function [22] and reviewed by VG to validate integrity of transcription.

### Objectives

#### Primary feasibility objectives

The primary outcome was the number of successful breath samples collected. Success was measured in terms of the following:

- Percentage of breath samples processed within 2 h
- Percentage of breath samples with %CO_2_ ≥ 3.5%
- Difference in patient-reported pain before and after breath collection
- User-reported ease of breath collection

#### Secondary objectives

The secondary objective was to explore the potential of using the VOC signatures to differentiate between patients with and without appendicitis.

### Sample size

Sample size was chosen to enable accurate estimation of the proportion of successful breath sample collection (*n*) and to provide a minimum number of appendicitis patients (*m*) for exploratory analysis. To ensure both objectives were met, patients were planned to be recruited until *n* ≥ 50 AND *m* ≥ 5. The number of cases was determined so that at least preliminary performance of the breath test could be derived. An estimated appendicitis incidence rate of 10% was based on unpublished baseline data from 2400 referred to our centre with suspected appendicitis. The expected sample size was 50 patients.

### Analysis

#### Primary feasibility outcomes

The rate of successful breath sample collection was calculated as the proportion of study participants from which we obtained a VOC signature. The difference in patient-reported pain (pain after − pain before) was assessed using a two-sided Wilcoxon signed rank test. Associations between difficulty of breath collection, patient age and pain after breath collection were visualised using pair-wise scatter plots, and Spearman correlation coefficients, *r*, were reported. 95% confidence intervals were estimated by converting *r* into a *z*-score using the Fisher transformation.

#### Secondary outcomes

The VOC signature of each participant was summarised into one value per sensor channel to avoid overfitting, by integrating each channel over time. We normalised the integrated VOC signatures to have a mean of zero and standard deviation of one. Due to a limited sample size, we fitted a logistic regression with L1 regularisation at strength 0.1 (i.e. a Lasso regression [23]) to model any association between the summarised VOC signatures and definitive appendicitis. Model performance was reported via a confusion matrix. In addition, precision, recall and F1 score (harmonic mean of precision and recall) of the model at a threshold of 0.5 were reported. Point estimates were calculated on the original data whilst 95% CIs were calculated using 1000 bootstrap samples.

Precision and recall are defined as [24]:

$$ \mathrm{Precision}=\frac{\mathrm{True}\ \mathrm{positives}}{\mathrm{True}\ \mathrm{positives}+\mathrm{False}\ \mathrm{positives}} $$

$$ \mathrm{Recall}=\frac{\mathrm{True}\ \mathrm{positives}}{\mathrm{True}\ \mathrm{positives}+\mathrm{False}\ \mathrm{negatives}} $$

We also reported an overall measure of model performance, the area under the receiver operator characteristic curve (AUROC) [25].

## Results

**Fig. 2:**
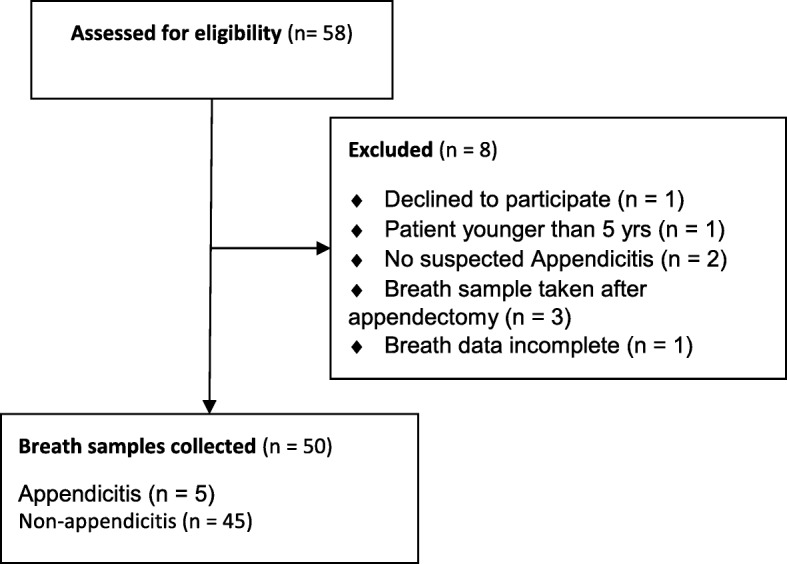
Study enrolment flow diagram

Fifty-eight participants were recruited to the study between August 2017 and January 2018. Of these, eight participants did not meet the inclusion criteria and were excluded from analysis (Fig. 2).

The primary diagnosis was unclear in two cases. One case was classified histologically as ‘peri-appendicitis’ with normal mucosa, and the other as inflammatory bowel disease. In both cases, there was evidence of inflammation at the appendix, but the cases were deemed not to be primary diagnosis appendicitis. Baseline clinical data are reported in Table 1.

**Table 1:** Baseline clinical data

	Non-appendicitis (*n* = 45)	Appendicitis (*n* = 5)
Sex		
Female	21	2
Male	24	3
Mean age in years (std)	10.6 (2.9)	9.9 (2.9)
Operated?		
Yes	2	5
No	43	0
Regular medication		
Yes	3	0
No	33	5
Not recorded	9	0
Antibiotics		
Yes	2	5
No	43	0

### Primary feasibility outcomes

Breath samples were collected from all 50 (100%) patients who met the study inclusion criteria, between August 2017 and January 2018.

Breath samples were processed within 2 h in 36/45 (80%) of patients. Of the 45, 44 (98%) were processed within 3 h. Processing time was not recorded for 5 participants.

The median difference in pain (scored 0–10) evaluated before and after the breath sample was 0 (IQR 0 to 0, range − 2 to 2); there was no significant change in reported pain using the two-sided Wilcoxon signed rank test (*p* = 0.49). For the five participants with confirmed appendicitis, two had a decrease in pain, one had an increase and two had no change in reported pain.

**Fig. 3:**
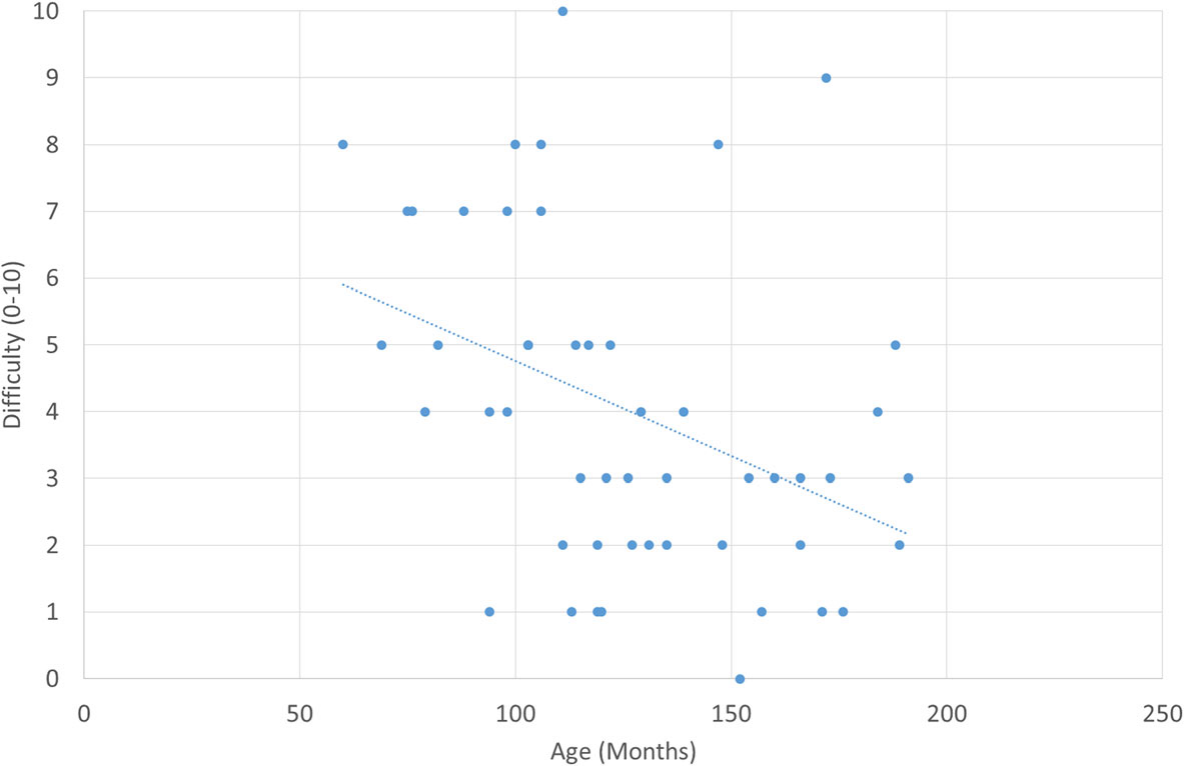
Negative correlation between age and difficulty of sample collection (*y* = 7.6–0.03*x*, Pearson *R* = − 0.396)

The median difficulty of sample collection (scored 0–10 by MI) was 4. There was moderate correlation between difficulty of collection and participant age (Spearman *r* = − 0.43, 95% CI − 0.60 to − 0.22, Fig. 3). There was no correlation between difficulty of collection and reported pain before the sample (Spearman *r* = − 0.11, 95% CI − 0.33 to 0.12), or between pain and participant age (Spearman *r* = 0.14, 95% CI − 0.09 to 0.36).

Breath samples had CO_2_ ≥ 3.5% (indicating alveolar breath) in 19/49 (39%) of patients. Of the 19, 3 were later confirmed to have had appendicitis. The mean age and standard deviation in the alveolar breath group was 11.1 years (s.d. = 2.9), in contrast to 10.3 years (s.d. = 2.8) for those that did not provide alveolar breath. The difference was not statistically significant (two-tailed *T* test *P* = .31). CO_2_ was not recorded for one participant.

### Secondary outcomes

Lasso regression [22] trained on the integrated VOC signatures of all included patients produced a model with six statistically significant parameters: 5 of the integrated sensor readings and a constant bias term. The full confusion matrix is given in Table 2, and results are further summarised in Table 3.

**Table 2:** Confusion matrix for the Lasso regression model

	Predicted negative	Predicted positive
No appendicitis	36	9
Appendicitis	1	4

**Table 3:** Classification table for the Lasso regression model

	Precision	Recall	F1 score	Support
No appendicitis	0.97	0.80	0.88	45
Appendicitis	0.31	0.80	0.40	5
Weighted average	0.91	0.80	0.83	

Classification table summarising the model that uses VOC signatures to predict appendicitis, with a weighted average of each column based on the number of positive and negative patients.

Thirteen of the 50 patients were predicted as having appendicitis (at a threshold of 0.5) of which 4 actually had appendicitis, giving a positive predictive value of 0.31 (95% CI = [0.31, 0.71]). The negative predictive value was 0.97 (95% CI = [0.96, 0.98]). The sensitivity of the model was 0.83 (95% CI = [0.71, 0.83]), and specificity was also 0.83 (95% CI = [0.73, 0.86]). The area under the receiver operator characteristic curve (AUROC) for this model was 0.83 (95% CI = [0.71, 0.83]).

One patient with appendicitis was misclassified; contemporaneous notes showed that the clinical researcher expressed doubt about the quality of the associated breath sample, but no further information was available. Furthermore, of the five positive cases, this sample had the lowest %CO_2_.

## Discussion

Exhaled breath tests are feasible in children aged 5–15 with abdominal pain. In all but one case, approached participants were recruited and breath was collected successfully. In the single case that consent was declined, the participant’s guardian withdrew the participant from the study before breath collection was attempted. Breath collection was not associated with increased reported pain. This extends results in similar exhaled breath condensate tests in children without pre-existing pain [26, 27].

The ability to collect samples was satisfactory when evaluated by our investigator. Although samples were successfully obtained in all cases, we identified an association between the age of the participant and lower difficulty in obtaining a breath sample. It seems likely that breath collection from children aged under 5 will be harder to achieve. Younger children have the highest risk of delayed diagnosis and perforation and may obtain most benefit from improvements to diagnosis [28]. The results highlight the potential utility of breath collection systems designed specifically for younger children.

Alveolar breath was only obtained in 39% of patients. The presence of alveolar breath was not associated with participant age or diagnosis of appendicitis.

Of the 45 samples for which sample turnaround time was recorded, 36 were processed within 2 h and 44 within 3 h. This indicates that test results can be made available within a clinically relevant timeframe. We note that the time to process samples depended on the workload of the research assistant and is therefore an upper-bound estimate of time required.

Exploratory data analysis used a logistic regression model to predict appendicitis cases. Whilst the sample size was not designed to determine test accuracy, initial results were promising, especially as the VOC sensors were not specifically designed to detect appendicitis. Four of 5 (80%) appendicitis cases were correctly classified, and 34/45 (76%) negative cases were also correctly classified. These figures are similar to those achieved using traditional biomarkers such as white cell count [29], though our small sample size means that direct comparison is not appropriate.

To the best of our knowledge, this is the first attempt to show that VOC biomarkers may have discriminatory power to diagnose appendicitis.

Two of 5 breath samples that corresponded to appendicitis cases did not contain alveolar breath. Of these, one had %CO_2_ = 3.47, very close to the threshold, and was classified correctly. The single misclassified case of appendicitis had a much lower %CO_2_, 2.79%. These results provide weak initial evidence to suggest that alveolar breath is necessary for the classification of appendicitis. Whilst limited resources precluded better quality control of breaths, we note that specific breath devices that measure %CO_2_ at the point of breath collection are available [30]. Future work should examine whether both non-alveolar and alveolar breath is adequate for diagnosing of appendicitis.

### Limitations

One potential limitation of this study is the use of subjective measures for measuring endpoints. A 0–10 scale was used to determine whether pain increased or decreased before and after breath sampling. This scale has been validated in cohorts with children as young as 6 years old, and no other popular scale has been validated in 5-year-olds [21, 30]. Differences in interpretation of pain by study participants may mean that this method can only accurately determine whether pain increased or decreased; it cannot be used to assess the magnitude of the change. Similarly, difficulty in sample collection was also measured on a subjective 0–10 scale. In this case, the use of a single researcher reduced inter-rater reliability. This means that comparisons are more reliable, but the absolute magnitude is not.

This study was not powered to assess the performance of VOC analysis in detecting appendicitis. Whilst the reported AUROC of 0.83 is much greater than for the null model (that is, random guessing), the figures are based on a very small number of positive appendicitis cases. Additionally, we used an array of sensors that were not specifically targeted towards likely appendicitis VOCs. Development of disease-specific sensors from mass spectrometry studies would likely improve overall accuracy.

The gold standard for diagnosis was appendix histology. This may underestimate the true number of positive cases if a patient was mis-diagnosed as non-appendicitis, but presented at another hospital if symptoms persisted. As our centre is a regional unit, the likelihood of this situation is minimal.

Finally, we did not correct for potential confounders such as the presence or absence of guarding and the presence or absence of a coryzal illness. Although we initially considered these variables, poor inter-rater reliability meant that we considered the data too poor for practical use. Follow-on work could consider longitudinal changes in VOC, particularly if an appendectomy had been performed. In our case, this was not possible due to limitations in resource.

### Generalisability

Our results show that bedside breath-test style tests are plausible in an acute paediatric setting. For the first time, we demonstrate that this is the case even for those experiencing pain. For VOC analysis specifically, we have demonstrated that data can be collected and analysed in a timely manner. Timeliness in this setting means two things. First, that VOC processing occurs before the biomarker signal degrades. Second, that processing is fast enough to influence clinical decisions. We have estimated 2 h as a reasonable period of time in which to obtain a result, and this was possible for most patients. Whilst our result is device-specific, the process of breath capture into temporary storage containers before analysis is typical [31].

Further work is required to examine clinical validity and clinical utility. Even if VOC analysis demonstrates the ability to differentiate between appendicitis and non-appendicitis, it must demonstrate improved outcomes in comparison with the current diagnostic techniques before introduction into clinical practice.

## Conclusion

Our pilot evaluation study showed that breath collection for VOC can be successfully and consistently collected in an acute paediatric setting. Results of exploratory data analysis to determine VOC analysis accuracy are promising. These data will inform further investigations using appendix-specific sensors, on a larger population, to confirm sensitivity and specificity [32].

## Data Availability

The datasets used and/or analysed during the current study are available from the corresponding author on reasonable request.

## Abbreviation

VOC: Volatile organic compounds
